# *Antirrhinum majus* L. flower extract inhibits cell growth and metastatic properties in human colon and lung cancer cell lines

**DOI:** 10.1002/fsn3.1924

**Published:** 2020-10-01

**Authors:** Jina Seo, Jungjae Lee, Hyi Young Yang, Jihyeung Ju

**Affiliations:** ^1^ Department of Food and Nutrition Chungbuk National University Cheongju Korea

**Keywords:** adhesion, cancer, growth, invasion, migration, snapdragon flower

## Abstract

Snapdragon **(** *Antirrhinum majus* L.) flowers are one of the most frequently used edible flowers in different preparations of foods and drinks. In this study, we examined inhibitory effects of snapdragon flower extract (SFE) against distinctive properties of cancer cells, stimulated growth, and activated metastasis, using H1299 lung cancer and HCT116 colon cancer cell lines. SFE treatment at 100–1,000 μg/ml for 24–72 hr resulted in a time‐ and dose‐dependent growth inhibition in H1299 and HCT116 cells. Cell cycle analysis and Annexin V staining assay further revealed that SFE caused cell cycle arrest at G2/M phase and induction of apoptosis, indicating the growth inhibition by SFE is attributed to its G2/M cell cycle‐arresting and apoptosis‐inducing activities. SFE dose‐dependently enhanced generation of intracellular reactive oxygen species (ROS) and reduced mitochondrial membrane potential in H1299 cells but had no effect on intracellular ROS levels in HCT116 cells, suggesting that the type of apoptosis induced by SFE in H1299 cells is different to that in HCT116 cells. Furthermore, SFE alleviated invasion, levels of matrix metalloproteinases, migration, and adhesion in H1299 and HCT116 cells. These results indicate that SFE not only inhibits cell growth by cell cycle arrest at G2/M and apoptosis induction but also alleviates metastatic properties such as invasion, migration, and adhesion in lung and colon cancer cells.

## Graphical abstract

Snapdragon flower extract not only inhibits cell growth by cell cycle arrest at G2/M phase and induction of apoptosis but also alleviates metastatic properties such as invasion, migration, and adhesion in human lung and colon cancer cells.

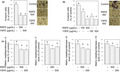

## 1 INTRODUCTION

Cancer is a major disease burden around the world. Among cancers at different organ sites, lung cancer is the most prevalent and leading cause of death in most countries, while colorectal cancer ranks second in both incidence and mortality in more developed countries (Siegel et al., 2019). Carcinogenesis is a multi‐stage process of cancer development, whereby normal cells evolve progressively to a tumorigenic and ultimately malignant state. Sustaining stimulated growth and activating metastasis are the most distinctive properties that cancer cells must acquire during the multi‐stage carcinogenesis (Hanahan & Weinberg, 2011). Stimulated growth is the fundamental characteristics of cancer cells, which is achieved by dysregulated cell cycle and resisted programmed cell death, called apoptosis. Metastasis, the major cause of treatment failure and the death of cancer patients, is the ultimate step of the multi‐stage carcinogenesis where secondary tumors are formed at the sites distant from the site of primary lesion (Hanahan & Weinberg, 2011; Siegel et al., 2019).

Diet has been recognized as a major determinant in the cancer etiology (Grosso et al., 2017). The most consistent finding on diet and cancer is an association between adequate consumption of plant‐based foods, such as fruits, vegetables, and whole grains, and reduced risk of several cancers, including cancers of digestive and respiratory tracts (Grosso et al., 2017). The benefits from consuming plant‐based foods have been attributed to different phytochemicals (Johnson, 2007). However, evidence that an isolated compound can entirely explain the cancer‐preventive activity of a particular plant‐based food has been insufficient (Liu, 2004). Therefore, it would be still important to investigate anticancer activities of plant‐based foods as a mixture containing a large number of different classes of phytochemicals.

Snapdragon **(** *Antirrhinum majus* L.) belongs to the family Plantaginaceae and is native to northern Africa and southern Europe (Al‐Snafi, 2015). The flowers and leaves of snapdragon have been used as traditional herbal medicine for treating several symptoms and diseases, including watery eyes, gum scurvy, hemorrhoids, ulcers, liver disorder, and tumors (Al‐Snafi, 2015). The flowers, particularly, are among the most popular edible flowers and frequently introduced in different preparations of foods and drinks, such as salad, desserts, soups, teas, and liquors, for decorative and flavor‐enhancing purposes (Rop et al., 2012). Despite rich uses of the flower in medicinal and food products, only a small number of studies have reported its antioxidant, antimicrobial, hemolytic, and wound‐healing activities (Al‐Snafi, 2015; Saqallah et al., 2018); information on other biological activities remains limited.

In the current study, we investigated the potential inhibitory effects of snapdragon flower extract (SFE) against stimulated growth and activated metastasis using two human cancer cell lines representing highly proliferative and metastatic properties (Li, Huang, et al., 2018; Wang et al., 2019), H1299 nonsmall cell lung carcinoma cells and HCT116 colorectal carcinoma cells. The results presented herein will be helpful to provide basic knowledge on the cancer‐inhibitory activities of SFE and scientific evidence for further development and application of functional food and medicinal products using SFE.

## 2 MATERIALS AND METHODS

### 2.1 Materials

RPMI and Macoy's 5A media were purchased from Gibco. Fetal bovine serum (FBS) was purchased from Thermo Scientific. Streptomycin and penicillin were purchased from Welgene Inc. Sodium carbonate, Folin–Ciocalteu's reagent, gallic acid, diethylene glycol, sodium hydroxide, quercetin, (+)‐catechin, dimethyl sulfoxide (DMSO), *N*‐acetylcysteine (NAC), 3‐[4,5‐dimethylthiazol‐2‐yl]‐2,5‐diphenyl tetrazolium bromide (MTT), RNase, propidium iodide (PI), 2′,7′‐dichlorofluorescein diacetate (DCFH‐DA), 3,3′‐dihexyloxacarbocyanine iodide (DiOC_6_), Matrigel, crystal violet, sodium dodecyl sulfate, and gelatin were purchased from Sigma‐Aldrich (St. Louis). Multi‐well plates were purchased from Corning Inc. All other chemicals and solvents (LC grade) were purchased from Fisher Scientifics.

### 2.2 Snapdragon flower extract

Snapdragon flowers were purchased from a local farm (Seoul, Korea), sorted by colors (red and yellow), and washed. Freeze‐dried (PH1316, IshinBioBase, Yangju, Korea) red and yellow flowers were separately ground into powder and extracted with 80% ethanol (40‐fold volume) for 6 hr at room temperature. After centrifugation at 1,500 *g* for 5 min (A320101, Gyrozen), the solvent was evaporated using a speed vacuum concentrator without additional heating applied (NB‐503CIR, N‐bioteck). The remaining dried extract of red and yellow flowers (RSFE and YSFE, respectively) was weighed to calculate the extraction yield and stored in deep freezer for further use.

### 2.3 Phytochemical compositions

The total polyphenol content was determined using Folin–Ciocalteu method (Margraf et al., 2015). Briefly, SFE reconstituted in ethanol (4 mg/ml), 8% sodium carbonate, distilled water, and Folin–Ciocalteu's reagent were mixed in a ratio of 6:20:10:3 (v/v) and then incubated for 30 min at room temperature. The resulting absorbance was read at the wavelength of 650 nm using a microplate reader (Bio‐Rad Laboratories). Total polyphenol content was calculated as mg gallic acid equivalent (GAE) per g of dried extract.

The total flavonoid content was determined according to previous report (Csepregi et al., 2013) with minor modification. Briefly, SFE reconstituted in ethanol (4 mg/ml), diethylene glycol, and 1 N sodium hydroxide were mixed in a ratio of 3:10:5 (v/v) and then incubated for 1 hr at room temperature. The resulting absorbance was read at the wavelength of 415 nm using a microplate reader (Bio‐Rad Laboratories). Total flavonoid content was calculated as mg quercetin equivalent (QE) per g of dried extract.

Proanthocyanidin content was determined according to previous report (Aastrup, 1985) with minor modification. Briefly, SE reconstituted in ethanol (4 mg/ml), 2% vanillin, and 8 N HCL was mixed in a 1:1:1 ratio (v/v). After incubation for 20 min at 37°C, the absorbance was read at the wavelength of 495 nm in a microplate reader (Bio‐Rad Laboratories). Proanthocyanidin content was calculated as mg (+)‐catechin equivalent (CE) per g of dried extract.

Carotenoid content was determined according to previous report (Scolnik et al., 1980). SFE was dissolved in DMSO, and the absorbance was read at 470 nm (*A*_470_), 647 nm (*A*_647_), and 663 nm (*A*_663_). Total carotenoid content was calculated by the equation of (1,000*A*_470_ + 333.4837*A*_663_ − 1,343.057*A*_647_) × 1.136 and shown as mg per g of dried extract.

### 2.4 Cell culture and general scheme of treatment

H1299 human lung cancer cells and HCT116 human colon cancer cells (Korean Cell Line Bank, Seoul, Korea) were cultured in RPMI and Macoy's 5A media, respectively, at 37°C with 95% humidity and 5% CO_2_. Each medium was premixed with 10% FBS, 0.1 mg/ml streptomycin, and 100 units/ml penicillin. DMSO was used as a vehicle to deliver SFE, and its final concentration was less than 0.2% (v/v) in all experiments. Serum‐free media were used to treat SFE unless indicated otherwise.

### 2.5 Cell viability

H1299 and HCT116 cells were seeded in 96‐well plates at a density of 1 × 10^4^ cells/well. After overnight incubation, cells were treated with SFE at 0, 100, 500, and 1,000 μg/ml for 72 hr. In another experiment, H1299 cells were pretreated with 2 mM NAC for 2 hr and then cotreated with 500 μg/ml SFE and 2 mM NAC for 72 hr. After aspiration of medium, cells were washed with PBS twice and then incubated with MTT at 0.5 μg/ml for 4 hr. The formazan dye formed by viable cells was solubilized by DMSO, and the absorbance was monitored at 540 nm using a microplate reader (Bio‐Rad Laboratories).

### 2.6 Cell cycle

H1299 and HCT116 cells at a density of 1 × 10^5^ cells/well were starved for 24 hr in serum‐free media and then treated with SFE at 500 µg/ml (for H1299) or 1,000 μg/ml (for HCT116) in serum‐complete media for 72 hr. Cells were trypsinized, washed with PBS, and fixed with 70% of methanol. Cells were incubated with 1 μg/ml RNase and 50 μg/ml PI for 30 min at 37°C. For each determination, 10,000 cells were counted, and cells at different phases such as sub‐G1, G1/G0, S, and G2/M were analyzed using flow cytometer (BD Biosciences).

### 2.7 Apoptosis

Apoptotic cells were quantified by Annexin V/PI double staining assay using apoptotic detection kit (BioVision). H1299 cells were seeded in 6‐well plates at a density of 1 × 10^5^ cells/well. After overnight incubation, cells were treated with SFE at 0 or 500 μg/ml in serum‐complete media for 72 hr. Cells were then gently detached by brief trypsinization, washed, and stained with annexin V‐FITC and PI for 10 min in the dark. The stained cells were analyzed using flow cytometer (FACS Calibur‐S System, BD Biosciences). Annexin V‐positive/PI‐negative cells and annexin V‐positive/PI‐positive cells were identified as early and late apoptotic cells, respectively.

### 2.8 Intracellular reactive oxygen species

H1299 and HCT116 cells were seeded in 96‐well black plates at a density of 3 × 10^5^ cells/well. After overnight incubation, cells were treated with SFE at 0, 100, 500, and 1,000 μg/ml for 72 hr. In another experiment, H1299 cells were pretreated with 2 mM NAC for 2 hr and then treated with 500 μg/ml SFE in the presence of NAC for 72 hr. After aspiration of medium, cells were incubated with 100 μM DCFH‐DA in the dark for 30 min at 37°C. Intracellular reactive oxygen species (ROS) oxidize DCFH‐DA to 2′,7′‐dichlorofluorescin (DCF), which was monitored at the excitation and emission wavelength of 485 nm and 528 nm, respectively, using a fluorescence microplate reader (FLx800, Biotek Instrument).

### 2.9 Mitochondrial membrane potential

H1299 cells were seeded in 6‐well plates at a density of 1 × 10^5^ cells/well. After overnight incubation, cells were treated with SFE at 0, 100, 500, and 1,000 μg/ml for 72 hr. After aspiration of medium, cells were then incubated with 100 nM DiOC_6_, a dye selective for the mitochondria of live cells, in the dark for 30 min at 37°C. Relative fluorescence intensity was measured using a flow cytometer (BD Biosciences).

### 2.10 Cell invasion and migration

Invasion and migration assays were performed using a transwell insert with 8 µm pore size (Sigma‐Aldrich). H1299 and HCT116 cells (1 × 10^5^ cells/well) containing SFE at 0–500 µg/ml in serum‐free media were loaded on the insert. For the invasion assay, the insert was precoated with Matrigel (20 µg/well) to simulate a human basement membrane. After 24 hr, invasive or migratory cells moving through the insert toward the outer well containing serum‐complete media were fixed with methanol for 10 min and stained with 0.1% crystal violet for 30 min. After gentle wash, the dye was dissolved by adding 1% sodium dodecyl sulfate and quantified spectrophotometrically using a plate reader (Bio‐Rad Laboratories) at the wavelength of 540 nm.

### 2.11 Matrix metalloproteinases

H1299 cells were seeded in 24‐well plates at a density of 1 × 10^5^ cells/well. After overnight incubation, cells were treated with SFE at 500 μg/ml for 24 hr. The media were collected and analyzed for matrix metalloproteinases‐2, ‐7, ‐9, and ‐10 levels using enzyme‐linked immunosorbent assay kits (Koma Biotech Inc.) following the manufacturer's instruction.

### 2.12 Cell adhesion

H1299 and HCT116 cells were suspended at a density of 2 × 10^4^ cells/well in serum‐complete media containing SFE at 1,000 μg/ml and plated into 96‐well plates precoated with 0.5% gelatin. After 2 hr, 0.5 mg/ml of MTT in the fresh media was added to cells adherent to gelatin. Gelatin is a hydrolyzed collagen and frequently used for mimicking major component of extracellular matrix (Plant et al., 2009). Further analysis was performed as described for the determination of cell viability.

### 2.13 Statistical analyses

All data were presented as the mean ± *SEM* of at least triplicates. Two‐tailed Student *t* test was used for comparing two groups. One‐way ANOVA followed by Duncan test was used for comparing more than two groups. Regression analysis was used for determining time‐ and dose‐dependent response. Significance was reached at *p* < .05.

## 3 RESULTS AND DISCUSSION

### 3.1 Phytochemical contents of SFE

A recent study has reported that the snapdragon flowers are rich in diverse phytochemicals (Gonzalez‐Barrio et al., 2018). To confirm the richness of phytochemicals, we first determined the content of major classes of phytochemicals, such as polyphenols, flavonoids, proanthocyanidins, and carotenoids, in SFE used in our study. Polyphenols, secondary metabolites widely distributed in higher plants, are a large class of compounds containing multiples of phenol units. Flavonoids, belonging to polyphenols, are characterized by two phenyl and one heterocyclic ring structure. Proanthocyanidins are an oligomeric flavonoid. Carotenoids are a class of naturally occurring pigments and structurally characterized by the presence of tetraterpenoids containing 8 isoprenes. These classes of phytochemicals are ubiquitously found in plants, and their wide range of health benefits, including cancer‐inhibitory activities, have been extensively studied (Krzyzanowska et al., 2010).

Total polyphenol and flavonoid contents of RSFE were 15.4 mg GAE/g and 43.2 mg QE/g, resp**e**ctively, which were similar to those of YSFE, 12.8 mg GAE/g and 48.1 mg QE equivalent/g, resp**e**ctively (Table 1). The total polyphenol content of SFE found in our study was similar to those of snapdragon flowers reported previously (10–28 mg GAE/g) (Gonzalez‐Barrio et al., 2018) and within the range of other edible flowers (Kaisoon et al., 2011; Li et al., 2014; Song et al., 2011). The total flavonoid contents found in our study were much higher than those of snapdragon flowers reported previously (1–2 mg CE/g) (Gonzalez‐Barrio et al., 2018) but within the range of other edible flowers (11–68 mg rutin equivalent/g) (Kaisoon et al., 2011). The differences in the contents are likely due to different botanical origins, extraction protocols, and other experimental conditions used for different studies. RSFE contained significantly higher proanthocyanidin levels (312.6 mg CE/g) but lower total carotenoid levels (8.6 mg/g) than YSFE (182.3 mg CE/g and 243.6 mg/g, respectively). These results were consistent with previous reports showing that the flowers with distinct colors, especially red, purple, or blue, contain high amounts of anthocyanidins, while the flowers with pale colors, like yellow or white, contain low amounts of anthocyanidins but high amount of carotenoids (Garzon et al., 2015; Vukics et al., 2008). Our results indicated the richness of phytochemicals in SFE, which allowed us to further evaluate its potential cancer‐inhibitory activities.

**TABLE 1:** Phytochemical content of SFE

Phytochemicals	RSFE	YSFE
Extraction yield (%)	23.1	32.4
Total polyphenols (mg gallic acid equivalent/g dried extract)	15.4 ± 0.8^†^	12.8 ± 0.8
Total flavonoids (mg quercetin equivalent/g dried extract)	43.2 ± 2.0	48.1 ± 2.6
Proanthocyanidins (mg catechin equivalent/g dried extract)	312.6 ± 13.6*	182.3 ± 30.6
Carotenoids (mg/g dried extract)	8.6 ± 1.8	243.6 ± 0.3***

Note

Asterisks mean difference between RSFE and YSFE (*p < .05, ***p < .001).

^†^ Mean ± *SEM* of ≥3 determinations.

### 3.2 SFE inhibited the growth of human lung and colon cancer cells

**FIGURE 1:**
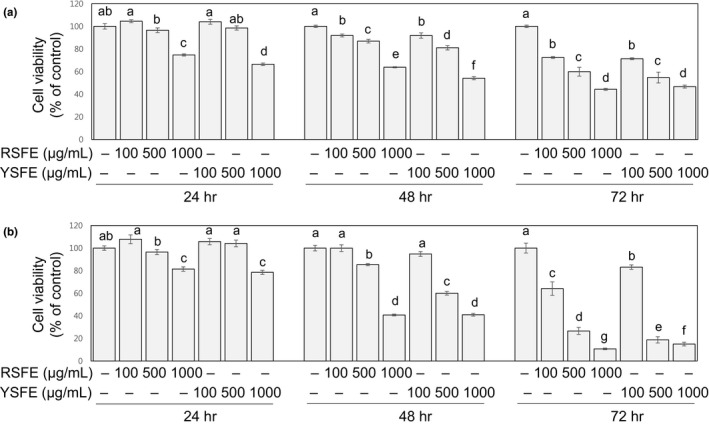
SFE suppressed the growth of H1299 human lung cancer cells and HCT116 human colon cancer cells. H1299 (a) and HCT116 cells (b) were treated with SFE (0–1,000 μg/ml) for 24 hr, 48 hr, and 72 hr, and viable cells were shown as % of control. Different letters (a‐g) indicate significance (*p* < .05)

Stimulated growth is the most essential trait of cancer cells (Hanahan & Weinberg, 2011). To determine the growth‐inhibitory activities of SFE in human lung and colon cells, cell viability assay was performed. As shown in Figure 1, the treatment with SFE at 100, 500, and 1,000 μg/ml for 24–72 hr inhibited the growth of H1299 and HCT116 cells in a time‐ and dose‐dependent manner (*R* ^2^ ≥ 0.9, *p* < .001). Particularly at the 72 hr time point, SFE at all concentrations, even at the lowest concentration (100 μg/ml), exhibited significant growth‐inhibitory effects in both H1299 (with estimated IC_50_ values of 800–820 μg/ml) and HCT116 cells (with estimated IC_50_ values of 260–305 μg/ml).

### 3.3 SFE induced cell cycle arrest and apoptosis in human lung and colon cancer cells

**FIGURE 2:**
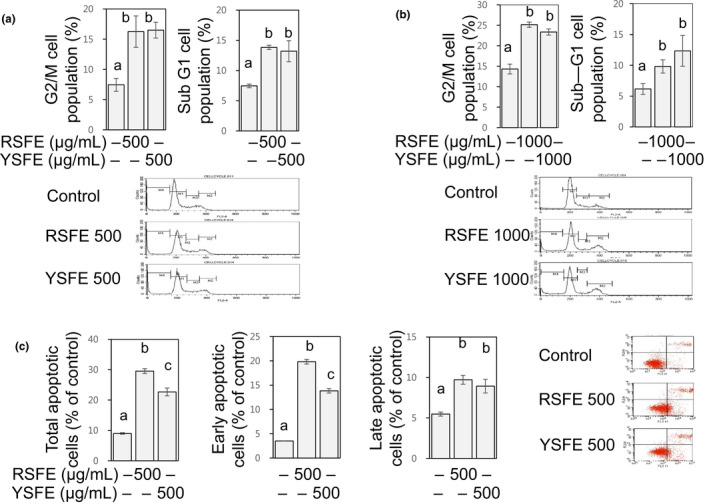
SFE induced G2/M cell cycle arrest and apoptosis in H1299 and HCT116 cells. H1299 (a) and HCT116 cells (b) were treated with SFE (500 µg/ml and 1,000 µg/ml, respectively) for 72 hr, and the cells at G2/M (M2) and sub‐G1 (M4) phases (%) were counted by flow cytometry. Additionally, H1299 cells were treated with SFE (500 µg/ml) for 72 hr, stained with Annexin V‐FITC and PI, and analyzed by flow cytometry (c). Total apoptotic cells were calculated by summing early (Annexin V+/PI−) and late apoptotic cells (Annexin V+/PI+). Different letters (a‐c) indicate significance (*p* < .05)

Uncontrolled cell cycle progression is a cardinal feature of cancer cells (Evan & Vousden, 2001; Hanahan & Weinberg, 2011). Apoptosis is a vital process for normal cell turnover. Defective apoptosis represents a major causative factor in the development and progression of cancer. Impediment of cell cycle progression and induction of apoptosis in cancer cells, therefore, are recognized as an effective preventive and therapeutic approach against cancers (Hanahan & Weinberg, 2011; Pan & Ho, 2008). To examine possible alteration in cell cycle distribution and induction of apoptosis by SFE, cell cycle analysis was performed. SFE significantly elevated the percentage of cell population at G2/M phase to 1.8‐fold to 1.9‐fold of the control H1299 cells (at 500 µg/ml) and to 1.6‐fold to 1.8‐fold of the control HCT116 cells (at 1,000 µg/ml) (Figure 2a,b), indicating G2/M arrest‐inducing activities of SFE. SFE also significantly elevated the percentage of cell population at sub‐G1 phase to 2.2‐fold of the control H1299 cells (at 500 µg/ml) and to 1.6‐fold to 2.1‐fold of the control HCT116 cells (at 1,000 µg/ml) (Figure 2a,b). Since the cells at sub‐G1 phase are apoptotic (Evan & Vousden, 2001), these results indicate apoptosis‐inducing activities of SFE. To confirm the induction of apoptosis by SFE, Annexin V/propidium iodine double staining assay was performed using H1299 cells. SFE at 500 μg/ml increased the percentage of both early and late apoptotic cells to 3.7‐fold to 5.5‐fold and 1.6‐fold to 1.8‐fold of the control cells, respectively, resulting in increased percentage of total apoptotic cells to 2.3‐fold to 3.3‐fold of the control (Figure 2c). Since dysregulated cell cycle and evaded apoptosis are important contributors to the stimulated growth of cancers (Evan & Vousden, 2001; Hanahan & Weinberg, 2011), our results suggest that the growth‐inhibitory activities of SFE found in this study (Figure 1) are attributed to the cell cycle arrest at G2/M and induction of apoptosis.

### 3.4 SFE enhanced intracellular ROS generation and reduced mitochondrial membrane potential in human lung cancer cells

**FIGURE 3:**
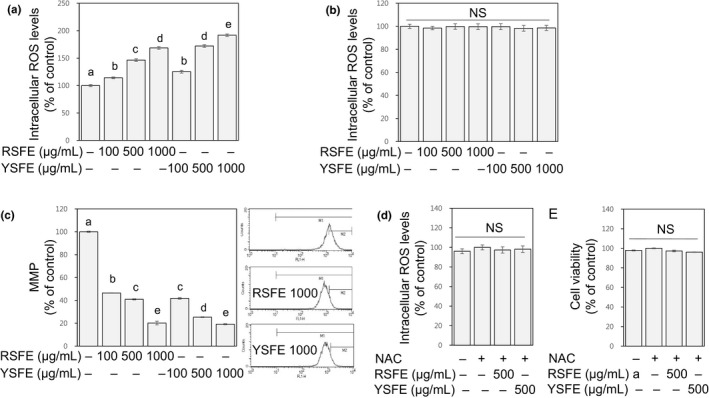
SFE enhanced intracellular ROS generation and reduced MMP in H1299 cells. H1299 (a, c) and HCT116 cells (b) were treated with SFE (0–1,000 μg/ml) for 72 hr, and intracellular ROS levels (a, b) and MMP (c) were present as % of control. H1299 cells were pretreated with NAC (2 mM) and then treated with SFE (500 μg/ml) in the presence of NAC for another 72 hr. Intracellular ROS levels (d) and cell viability (e) were present as % of control. Different letters (a‐e) indicate significance (*p* < .05)

Cells induce apoptosis by sensing different stress stimuli, including ROS. Mitochondria play an integral role in generating endogenous ROS by incomplete metabolism of consumed oxygen and electron leakage. Mitochondrial ROS induces sequential events, including collapse of mitochondrial membrane potential, and further activates intrinsic apoptotic signaling (Lowe & Lin, 2000). Increased production of intracellular ROS and disruption of mitochondrial membrane integrity, therefore, are regarded as early events in mitochondrial intrinsic apoptosis (Marchi et al., 2012; Redza‐Dutordoir & Averill‐Bates, 2016). To determine whether SFE‐treated cells were subjected to intracellular ROS‐mediated mitochondrial intrinsic apoptosis, intracellular ROS levels, and mitochondrial membrane potential (MMP) were measured. The treatment of H1299 cells with SFE at 100, 500, and 1,000 μg/ml for 72 hr resulted in increased intracellular ROS levels and decreased MMP in a dose‐dependent manner (*R* ^2^ ≥ 0.9, *p* < .001, Figure 3a,c). The increases in intracellular ROS level, however, were not found in HCT116 cells at 72 hr time point (Figure 3b) and other earlier time points (24 hr and 48 hr; data not shown). These results suggest that the type of apoptosis induced by SFE in H1299 cells is different to that in HCT116; the SFE‐induced apoptosis in H1299 cells is mitochondria‐mediated intrinsic while the SFE‐induced apoptosis in HCT116 cells is not mediated by intracellular ROS.

To further investigate the role of SFE‐induced intracellular ROS in the inhibition of H1299 cell growth, cells were treated with SFE at 500 μg/ml in the presence of NAC, a cell‐permeable ROS scavenger. NAC completely prevented the SFE‐induced elevation of intracellular ROS and inhibition of cell growth (Figure 3d,e). These results suggest that SFE‐induced intracellular ROS is responsible for the SFE‐induced inhibition of H1299 cell growth. Cancer cells are not capable of adapting to oxidative stress due to decreased capacity for ROS metabolism, and therefore, pro‐oxidants exacerbating oxidative stress in cancer cells often exert cytotoxic effects or enhance the efficacy of chemotherapeutic agents (Schumacker, 2006). Many phytochemicals have been shown to exhibit both antioxidant and pro‐oxidant activities. The mode of action may depend on the extent of oxidative stress, the concentrations of compounds, and the types of cancers (Chikara et al., 2018). Further studies are needed to verify whether such pro‐oxidant effects of SFE found in H1299 cells are still responsible for the growth‐inhibitory effects of SFE against lung carcinogenesis in vivo.

### 3.5 SFE inhibited invasion in human lung and colon cancer cells

**FIGURE 4:**
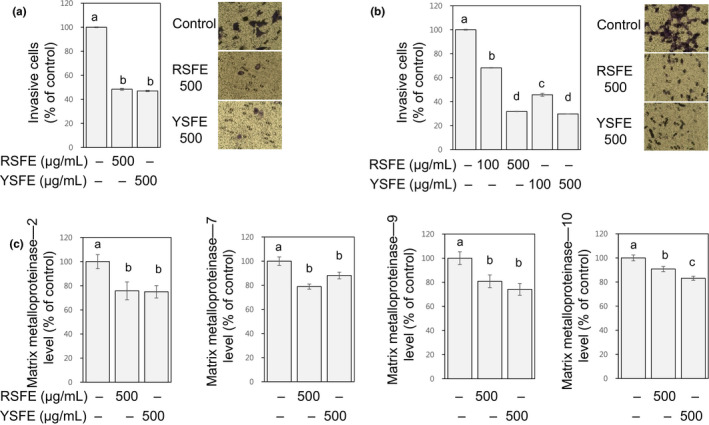
SFE inhibited invasion in H1299 and HCT116 cells. H1299 (a, c) and HCT116 (b) cells were treated with SFE (0–500 μg/ml) for 24 hr. Invasive cells (a, b) and matrix metalloproteinases‐2, 7, 9, and 10 levels (c) were shown as % of control. Different letters (a‐d) indicate significance (*p* < .05)

Invasion is a process of cells grown in the primary tumor penetrating into surrounding stroma, which is a critical step of cancer metastasis (Hanahan & Weinberg, 2011). Matrix metalloproteinases, a family of zinc‐dependent endoproteases degrading various proteins in the extracellular matrix, act as major contributors in the invasion and further malignant progression during metastasis (Conlon & Murray, 2019). Treatment with SFE significantly inhibited invasion of H1299 (to 45%–47% of the control, at 500 μg/ml) and HCT116 cells (to 30%–68% of the control, at 100–500 μg/ml) in a Matrigel‐coated transwell assay (Figure 4a,b). Concomitantly, SFE decreased levels of matrix metalloproteinase‐2, ‐7, ‐9, and ‐10 in H1299 cells (to 74%–90% of the control, at 500 μg/ml, Figure 4c). Matrix metalloproteinase‐2 and −9 are categorized into gelatinases which major substrate are gelatin and type IV collagen. Matrix metalloproteinase‐7 is a membrane‐type matrilysin degrading fibronectin, laminin, type VI collagen, and gelatin. Matrix melloproteinase‐10 is a stromelysin with a broad range of substrates (Conlon & Murray, 2019). Our results indicate that SFE possesses inhibitory activities against invasion, and such activities appear to be associated with suppressed levels different matrix metalloproteinases.

### 3.6 SFE alleviated migration and adhesion of human lung and colon cancer cells

**FIGURE 5:**
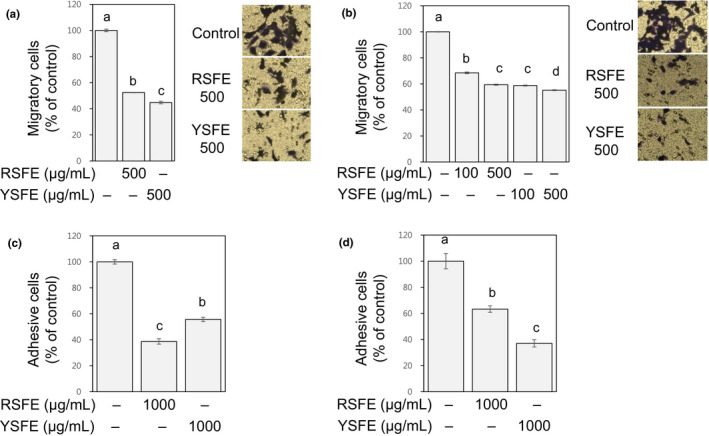
SFE alleviated migration and adhesion in H1299 and HCT116 cells. H1299 (a) and HCT116 cells (b) were treated with SFE (0–500 μg/ml) for 24 hr, and migratory cells were quantified as % of control. H1299 (c) and HCT116 cells (d) suspended in media containing SFE (1,000 μg/ml) were plated into gelatin‐coated 96‐well plate, and after 2 hr, adherent cells were quantified as % of control. Different letters (a‐d) indicate significance (*p* < .05)

Migration of cancer cells through extracellular matrix to reach blood or lymph vessels and alteration in adhesion of cell‐extracellular matrix are inevitable events during cancer metastasis (Hanahan & Weinberg, 2011). In both H1299 and HCT116 cells, SFE substantially alleviated migration (to 45%–68% of the control, at 100–500 μg/ml) and adhesion to gelatin (to 37%–63% of the control, at 1,000 μg/ml) (Figure 5). YSFE is slightly more effective than RSFE in inhibiting migration consistently in both H1299 and HCT116 cells (Figure 5a,b). Since lutein, a major carotenoid found in Snapdragon flowers (Gonzalez‐Barrio et al., 2018), is presumably present in the extract used in our study (Craft & Soares, 1992) and has been reported to inhibit migration of breast cancer cells ( Li, Zhang, et al., 2018), the greater activities of YSFE than HSFE may be ascribed to lutein contained in YSFE; further study is needed.

The inhibitory activities of SFE against invasion (at 24 hr time point, Figure 4), migration (at 24 hr time point, Figure 5a,b), and adhesion (at 2 hr time point, Figure 5c,d) were observed at noncytotoxic conditions where significant changes in cell viability were not found in SFE‐treated cells (Figure 1). Therefore, these activities are likely independent of the growth‐inhibitory activity of SFE. Our results (Figures 4 and 5) collectively suggest the antimetastatic activities of SFE against lung and colon cancer cells.

To the extent of our knowledge, this is the first study demonstrating inhibitory effects of SFE against cell growth and metastatic properties of human cancer cells. Further studies are warranted to standardize SFE and then verify whether such effects are demonstrated in laboratory animals and finally humans. Since a recent study has reported that snapdragon flowers are rich in different bioactive phytochemicals, including flavonol glycosides and anthocyanin (Gonzalez‐Barrio et al., 2018), it needs to be clarified whether the inhibitory activities of SFE observed in the present study are ascribed to an isolated single constituent or more than one constituents in combination. Detailed molecular mechanisms of the inhibitory action of SFE against lung and colon cancers also remain to be elucidated.

## 4 CONCLUSION

SFE effectively not only inhibits cell growth by cell cycle arrest at G2/M phase and induction of apoptosis but also alleviates metastatic properties such as invasion, migration, and adhesion in lung and colon cancer cells. These results will be helpful for understanding health benefits of SFE and developing relevant medicinal and functional food products.

## CONFLICT OF INTEREST

The authors declare that there is no conflict of interests.

## ETHICAL APPROVAL

This study does not involve any human or animal studies.
